# Number of parity/live birth(s) and cardiovascular disease among Iranian women and men: results of over 15 years of follow-up

**DOI:** 10.1186/s12884-020-03499-2

**Published:** 2021-01-07

**Authors:** Seyyed Saeed Moazzeni, Hossein Toreyhi, Samaneh Asgari, Fereidoun Azizi, Fahimeh Ramezani Tehrani, Farzad Hadaegh

**Affiliations:** 1 Prevention of Metabolic Disorders Research Center, Research Institute for Endocrine Sciences, Shahid Beheshti University of Medical Sciences, Tehran, Iran; 2 Endocrine Research Center, Research Institute for Endocrine Sciences, Shahid Beheshti University of Medical Sciences, Tehran, Iran; 3 Reproductive Endocrinology Research Center, Research Institute for Endocrine Sciences, Shahid Beheshti University of Medical Sciences, Tehran, Iran

**Keywords:** Cardiovascular disease, Parity, Live birth, The Tehran lipid and glucose study

## Abstract

**Background:**

Most previous studies conducted in non-Middle Eastern populations have suggested that an increase in the number of parity/live birth(s) leads to cardiovascular disease (CVD) development, although their findings were inconclusive on this issue for both sexes. Biologic and socioeconomic pathways were suggested to explain this association. We studied this issue among urban Iranian men and women.

**Methods:**

In this population-based cohort study, which included 3929 women and 2571 men aged ≥30 years, data for the number of parity/live birth(s) were obtained by a standard questionnaire. Participants were then annually followed for CVD events. Multivariable Cox proportional hazard models were used to estimate hazard ratios (HRs) and 95% confidence intervals (CIs) for the number of parity/live birth(s) and other traditional CVD risk factors.

**Results:**

During more than 15 years of follow-up, 456 and 524 CVD events have occurred among women and men, respectively. Among women, a J-shaped association was found between the number of live births and incident CVD with the lowest risk for women with two live births. Among women in multivariable analyses, each unit increase in parity had a HR of 1.05 (CI: 1.01–1.10), and having ≥4 parity was associated with a HR of 1.86 (0.97–3.56, *p*-value = 0.061). Among men, in comparison with participants who had 1 child, multivariable HRs of having 2, 3, and ≥ 4 children were 1.97 (1.24–3.12), 2.08 (1.31–3.31), and 2.08 (1.30–3.34), respectively.

**Conclusion:**

To the best of our knowledge, the current study is the first report on this issue in the Middle East and North Africa region, a region with a high burden of CVD. It can now be suggested that the number of parity/live birth(s) is linked to CVD among the Iranian population, with this issue being more prominent among men. Further research is needed to support our results and clarify the pathways between the number of parity/live birth(s) and CVD development among Iranian populations by considering potential risk factors, especially psycho-socio-economic risk factors.

**Supplementary Information:**

The online version contains supplementary material available at 10.1186/s12884-020-03499-2.

## Background

According to the latest GBD (Global Burden of Disease) data, cardiovascular disease (CVD) is the most common non-communicable disease, responsible for 17.8 million deaths in 2017 [1]. Likewise, two consequences of CVD (stroke and coronary heart disease (CHD)) are the leading causes of mortality around the world [2, 3]. Statistics show that the effects of this phenomenon in the Middle East and North Africa (MENA) region are more prominent [2, 4]. In this region, Iran, while reporting modest mortality from CVD, is among the countries with the highest prevalence of CVD, with an approximate prevalence of 11% (7.7% for CHD) [2, 5].

In addition to traditional modifiable CVD risk factors such as obesity, hypertension, hypercholesterolemia, diabetes, and smoking, it was shown that some reproductive factors could have a role in CVD development [6]. Pregnancy, as one of the most important events during a women’s life, is associated with several cardiometabolic changes, including weight gain, dyslipidemia, increased plasma glucose, and insulin resistance. While these kinds of changes are beneficial to both mother and fetus, they can also increase the prevalence of potential CVD risk factors. Additional stressors during pregnancy such as endothelial dysfunction, inflammation, and hemostasis process may also exacerbate this situation [7–9]. Results of previous studies on the association of parity (i.e., number of pregnancies reaching viable gestational age) and gravidity (i.e., number of times a woman is or has been pregnant, regardless of the pregnancy outcome) with the risk of CVD are inconclusive [9, 10]. Peters et al. showed that among 10 European countries, parous women had a 19% higher risk of CHD than nulliparous women, with this risk reaching up to 95% among women who had ≥5 children [10]. On the other hand, some others reported no significant association between parity and CVD [11–13]. Moreover, a J-shaped relationship was reported between parity and CVD among a Swedish female population, in which women with 2 births had the lowest risk of CVD, as compared to nulliparous and other parous women [8].

In addition to biomedical factors, some socioeconomic and lifestyle factors, which have a two-way causal relationship with the number of children (family size), can play a role in CVD development [14, 15]. These include social and economic characteristics, family and social supports, educational level, employment, income, lifestyle etc. This socioeconomic and lifestyle pathway is applicable not only to mothers but also to fathers. A few previous studies examined the effect of the number of live births on CVD development among men; however, this issue is controversial among men as well [16, 17]. A large study conducted on a Chinese population reported that having more children was associated with CVD outcomes among men [17]. On the other hand, Eisenberg et al. found a negative association between the number of children and cardiovascular mortality among American men [16].

To the best of our knowledge, most previous studies related to these issues have been conducted in developed and Western countries, and there is a lack of information on the effect of the number of parity/live birth(s) on CVD development in the MENA region, a region with a high burden of CVD [2, 4]. The aim of the current study is to determine whether the number of parity/live birth(s) is an independent risk factor for incident CVD among Iranian women and men aged ≥30 years, over 15 years of follow-up. Data was collected from the oldest cohort of the MENA region, namely the Tehran Lipid and Glucose Study (TLGS).

## Methods

### Study design and study population

This study was conducted within the framework of TLGS, which is a community-based cohort study on a representative sample of Tehran’s residents. TLGS pursues aims to determine the prevalence and incidence of non-communicable diseases and their risk factors and also prevent these diseases by advancing healthier lifestyles. TLGS enrollment has been conducted in two phases; phase one (January 31, 1999- July 03, 2001) and phase two (October 20, 2001- September 22, 2005). Data collection is planned to continue for at least 20 years at approximately 3-year intervals. More details of TLGS design and enrollment have been reported elsewhere [18].

A total of 5223 women (phase I=4459, phase II=764) and 4330 men (phase I=3468, phase II=862)aged ≥30 years were enrolled. Participants were excluded if they were single (194 women and 179 men), with prevalent CVD at baseline (281 women and 318 men), and with no live births (21 women and 8 men), leading to a total of 4727 women and 3825 men. Moreover, other exclusions included those with missing data on the number of parity/live birth(s) (198 women and 1045 men), missing information on covariates including body mass index (BMI), fasting plasma glucose (FPG), total cholesterol (TC), systolic/diastolic blood pressure (SBP/DBP), education level, smoking status, family history of premature CVD, history of miscarriage, and history of oral contraceptive pill (OCP) use (198 women and 177 men, considering overlap features in the number of missing data between covariates). Also, subjects with no follow-up data (402 women and 32 men) were excluded. Finally, 3929 women and 2571 men were eligible for our analysis.

### Clinical and laboratory measurements

Using a structured questionnaire, a trained nurse collected data for demographic data, past medical history, drug history, family history of premature CVD, education level, marital status, smoking habits, and physical activity level. Questionnaires also included data on the history of miscarriage and the number of parity/live birth(s).

According to TLGS setting [18], measurements of weight and height were done with shoes removed and wearing light clothing. Weight was measured to the nearest 100 g. The height of individuals was measured by a tape meter in a standing position. After a 15-min rest in a sitting position, two measurements of SBP and DBP were taken on the right arm. The mean of two measurements was defined as the subject’s blood pressure (BP).

On the day of blood collection, after 12 to 14 h of overnight fasting, a blood sample was collected between 7:00 and 9:00 AM from all participants in the TLGS research laboratory. Samples were analyzed on the same day. Details for laboratory measurements, including FPG, TC, high-density lipoprotein cholesterol (HDL-C), and triglycerides (TG) are published elsewhere [18].

### Outcome assessment

CVD data collection has been described in detail elsewhere [18]. Briefly, a trained nurse telephoned all participants annually and asked them about any cardiovascular events that had occurred during the past year. For all reported events, a home visit was made by a trained physician; the physician collected data from medical documents or death certifications (in case of mortality). Finally, the outcome committee, which included an internist, an endocrinologist, a cardiologist, and an epidemiologist, evaluated the outcome data and adjudicated events. A CHD event included cases of unstable angina pectoris (new cardiac symptoms or changing symptom patterns and positive ECG findings with normal biomarkers), angiographic-proven CHD, definite myocardial infarction (MI) (diagnosed by electrocardiogram (ECG) and biomarkers), probable MI (positive ECG findings and cardiac symptoms plus missing biomarkers or positive ECG findings plus equivocal biomarkers), and cardiac death (any death in the hospital due to CHD based on the above-mentioned criteria, or sudden cardiac death by cardiac disease happening ≤1 h after symptoms initiation according to verbal autopsy files). CVD events was a combination of any CHD event, fatal or non-fatal stroke (defined as a new neurological deficit lasting ≥24 h), and cerebrovascular death.

### Definition of terms

Type 2 diabetes mellitus (T2DM) was defined as one of these two criteria: i) FPG ≥7 mmol/L or ii) using glucose-lowering medications. Having TC ≥5.18 mmol/L or a history of lipid-lowering medications usage considered hypercholesterolemia. Hypertension was defined as either these three criteria: i) SBP ≥140 mmHg, ii) DBP ≥90 mmHg or iii) using antihypertensive medications. Smoking habit was categorized into three groups: i) current smoker, ii)former smokers and iii) never smokers. Education levels were classified into three categories: i) illiterate/primary school (reference group), ii) below diploma/diploma, and iii) above diploma. A history of CHD/stroke in a male first-degree relative aged < 55 years or female first-degree relative aged < 65 years was defined as a positive family history of premature CVD. For participants who were enrolled in phase I, the Lipid Research Clinic (LRC) questionnaire was used [19]; being physically active less than 3 days per week was considered as low physical activity. Using the Modifiable Activity Questionnaire (MAQ) for participants who were enrolled in phase II, subjects with < 600 MET (metabolic equivalent task-minutes per week) were in the low physical active group [18, 20, 21]. Parity was defined as the number of live childbirth plus the number of stillbirth (defined as the birth of an infant which died in the mother’s uterus after 20 weeks of gestation). A loss of an embryo or fetus before the 20th week of pregnancy defined as miscarriage.

### Statistical analyses

All analyses were done separately for each sex. Baseline characteristics across the number of live births (1, 2, 3, and ≥ 4) are expressed as mean ± standard deviation (SD) for continuous and number (%) for categorical variables. To compare baseline characteristics among different groups, the ANOVA test (or Kruskal–Wallis test for skewed variables) was employed for continuous variables. The Chi-square test was also applied for categorical ones.

To be able to capture a potential nonlinear association between the number of live births and incident CVD, restricted cubic splines with 4 knots, which defined the 5th, 25th, 75th, and 95th percentile, were used. This method enabled us to modify the model across the number of live births while considering a dose-response relationship [22]. Cox proportional hazard models were applied to evaluate the association of the number of parity/live birth(s) with incident CVD in two models: model 1 adjusted with baseline measurements of age; model 2 further adjusted with BMI, T2DM, hypertension, hypercholesterolemia, educational levels, smoking status, and family history of premature CVD for both sexes, as well as history of miscarriage and OCP use for women. The hazard ratios (HRs) and 95% confidence intervals (95% CI) were reported for the number of parity/live birth(s) and other CVD risk factors. The time to event was defined as either the time of censoring or the event occurring, whichever came first. We censored participants in the case of not-CVD causes of death, leaving the district or being in the study until 20 March 2016, without any event. Moreover, we rerun our data analysis using age as time axis, rather than applied as a confounder in a sensitive analysis.

For the Cox models, the proportionality was assessed with the Schoenfeld residual test. All proportionality assumptions were appropriate. Statistical analyses were performed using STATA version 14 (StataCorp LP, College Station, Texas) statistical software. For defining statistical significance, *P*-values < 0.05 were considered.

## Results

Baseline characteristics according to the number of live births are presented in Table 1 for both sexes. Generally, among continuous variables, cardiometabolic risk profiles became worse with increases in the number of live births. Thus, having more live births was associated with older age, higher BMI (only among women), increased BP, and higher levels of FPG, TC, and TG (only among women). Similarly, among categorical variables, having more live births was associated with higher prevalence of T2DM, hypercholesterolemia, and hypertension as well as higher use of glucose-lowering, lipid-lowering (only among women) and antihypertensive medications. Furthermore, a positive history of miscarriage became more prevalent with increases in the number of live births. Finally, as the number of live births increased, the percentage of those with a higher degree of education levels decreased significantly.

**Table 1:** Baseline characteristics according to the number of live births among women and men: Tehran Lipid and Glucose Study, Iran, 1999–2016

	Women					Men				
Number of Live births	1	2	3	4 ≤	***P***-value*	1	2	3	4 ≤	***P***-value*
**Number of participants**	305	906	906	1812		376	770	576	849	
**Continuous variables, Mean ± SD**										
Age (year)	38.2 ± 9.6	38.8 ± 7.5	44.0 ± 9.2	54.0 ± 9.9	< 0.001	37.3 ± 8.0	41.6 ± 7.7	48.7 ± 9.2	58.8 ± 9.2	< 0.001
BMI (kg/m^2^)	27.0 ± 4.3	27.6 ± 4.6	28.7 ± 4.5	29.6 ± 4.7	< 0.001	26.1 ± 4.0	26.0 ± 3.6	26.3 ± 4.0	26.4 ± 3.8	0.214
SBP (mmHg)	112.0 ± 16.2	112.6 ± 14.7	118.5 ± 17.4	129.3 ± 21.9	< 0.001	115.5 ± 14.7	115.9 ± 15.4	120.3 ± 18.1	128.6 ± 20.7	< 0.001
DBP (mmHg)	74.6 ± 9.8	75.9 ± 9.7	78.7 ± 10.5	81.3 ± 11.0	< 0.001	77.0 ± 11.0	77.0 ± 10.5	79.0 ± 11.5	80.2 ± 11.9	< 0.001
FPG (mmol/L)	5.2 ± 1.6	5.1 ± 1.3	5.4 ± 1.6	6.1 ± 2.5	< 0.001	5.2 ± 0.9	5.2 ± 1.2	5.6 ± 1.9	5.9 ± 2.2	< 0.001
TC(mmol/L)	5.2 ± 1.1	5.4 ± 1.1	5.6 ± 1.1	6.0 ± 1.3	< 0.001	5.2 ± 1.1	5.3 ± 1.1	5.4 ± 1.1	5.4 ± 1.1	0.002
HDL-C (mmol/L)	1.2 ± 0.3	1.2 ± 0.3	1.1 ± 0.3	1.2 ± 0.3	0.349	1.0 ± 0.3	1.0 ± 0.2	1.0 ± 0.3	1.0 ± 0.2	0.128
TG (mmol/L)	1.37 (1.10)	1.45 (1.05)	1.70 (1.21)	1.97 (1.31)	< 0.001	1.82 (1.42)	1.82 (1.27)	1.84 (1.44)	1.85 (1.29)	0.811
**Categorical variables, n (%)**										
Smoking status					< 0.001					< 0.001
Never	281 (92.1)	834 (92.1)	854 (94.3)	1676 (92.5)		188 (50)	391 (50.8)	302 (52.4)	483 (56.9)	
Former	6 (2.0)	12 (1.3)	9 (1.0)	66 (3.6)		39 (10.4)	121 (15.7)	81 (14.1)	165 (19.4)	
Current	18 (5.9)	60 (6.6)	43 (4.7)	70 (3.9)		149 (39.6)	258 (33.5)	193 (33.5)	201 (23.7)	
Educational levels					< 0.001					< 0.001
Illiterate/primary school	56 (18.4)	144 (15.9)	334 (36.9)	1442 (79.6)		41 (10.9)	96 (12.5)	154 (26.7)	521 (61.4)	
Below diploma/diploma	182 (59.7)	655 (72.3)	526 (58.1)	346 (19.1)		253 (67.3)	505 (65.6)	302 (52.4)	268 (31.6)	
Above diploma	67 (22.2)	107 (11.8)	46 (5.1)	24 (1.3)		82 (21.8)	169 (21.9)	120 (20.8)	60 (7.1)	
Low physical activity, yes	192 (63.0)	560 (61.8)	622 (68.7)	1283 (70.8)	< 0.001	273 (72.6)	574 (74.5)	408 (70.8)	585 (68.9)	0.083
T2DM, yes	15 (4.9)	33 (3.6)	66 (7.3)	316 (17.4)	< 0.001	11 (2.9)	30 (3.9)	64 (11.1)	117 (13.8)	< 0.001
Hypercholesterolemia, yes	146 (47.9)	471 (52.0)	560 (61.8)	1347 (74.3)	< 0.001	187 (49.7)	410 (53.2)	321 (55.7)	497 (58.5)	0.022
Hypertension, yes	27 (8.9)	115 (12.7)	196 (21.6)	744 (41.1)	< 0.001	52 (13.8)	109 (14.2)	130 (22.6)	281 (33.1)	< 0.001
Family history of premature CVD, yes	55 (18.0)	172 (19)	165 (18.2)	322 (17.8)	0.896	60 (16.0)	126 (16.4)	80 (13.9)	98 (11.5)	0.031
Glucose-lowering medication, yes	7 (2.3)	16 (1.8)	24 (2.6)	182 (10.0)	< 0.001	3 (0.8)	15 (1.9)	15 (2.6)	52 (6.1)	< 0.001
Lipid-lowering medication, yes	7 (2.3)	20 (2.2)	33 (3.6)	144 (7.9)	< 0.001	6 (1.6)	10 (1.3)	14 (2.4)	22 (2.6)	0.236
Antihypertensive medication, yes	12 (3.9)	41 (4.5)	61 (6.7)	330 (18.2)	< 0.001	5 (1.3)	17 (2.2)	22 (3.8)	56 (6.6)	< 0.001
OCP use, yes	18 (5.9)	90 (9.9)	63 (7.0)	43 (2.4)	< 0.001	–	–	–	–	–
History of miscarriage, yes	75 (24.6)	292 (32.2)	314 (34.7)	776 (42.8)	< 0.001	–	–	–	–	–

*SD* Standard deviation, *BMI* Body mass index, *SBP* Systolic blood pressure, *DBP* Diastolic blood pressure, *FPG* Fasting plasma glucose, TC Total cholestrol, *HDL-C* High density lipoprotein cholesterol, TG Triglycerides, *T2DM* Type 2 diabetes mellitus, *CVD* Cardiovascular disease, *OCP* Oral contraceptive pill. Values are shown as Mean ± SD and number (%), for continuous and categorical variables, respectively; for TG values are shown as Median (interquartile range)

*The comparison *p*-value between groups was calculated using ANOVA test for normal continues variables, Kruskal–Wallis test for skewed variables, and chi-square test for categorical variables

Among women, incident CVD occurred in 456 (11.6%) women during a median (interquartile range: IQR) follow-up of 15.9 (11.7–16.5) years. Among men, the incident CVD occurred in 524 (20.4%) men during a median (IQR) follow-up of 15.7 (11.3–16.4) years.

**Fig. 1:**
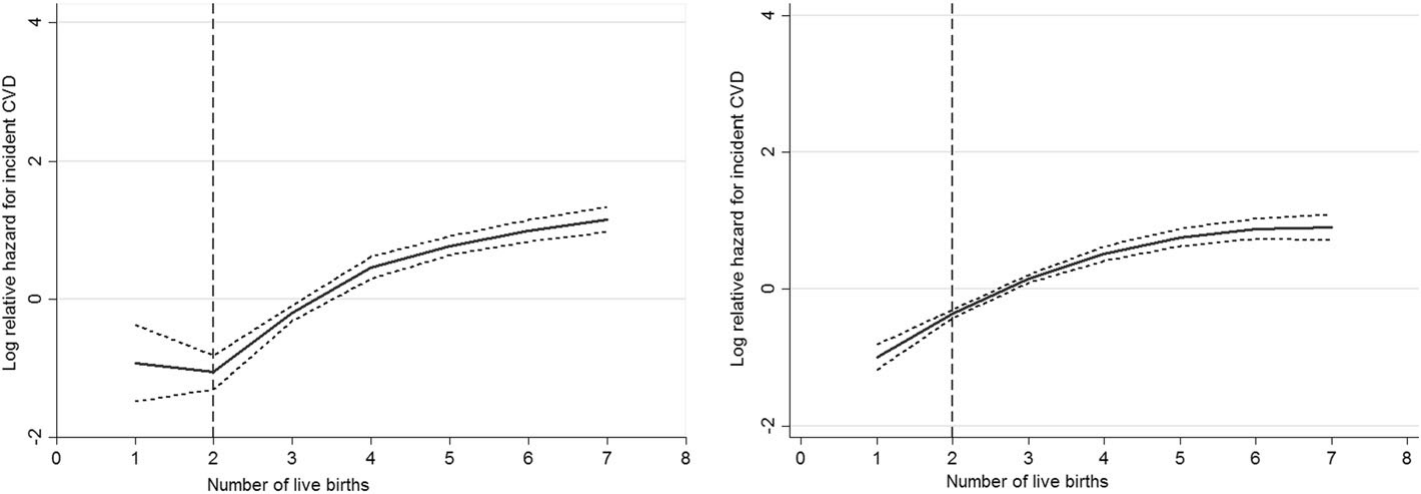
Restricted cubic spline curve for association (95% confidence interval) of the number of live births with incident cardiovascular disease (CVD) among Tehranian women and men: Tehran Lipid and Glucose Study, Iran, 1999–2016

As shown in Fig. 1, we rejected the null hypothesis that CVD risk was a linear function of the mean number of live births. Among women, the associations between the number of live births and the risk of incident CVD were J-shaped. The association of the number of live births with incident CVD appeared to decrease until the number reached two live births. After this point, the association appeared to rebound. Furthermore, the association was non-linear among men.

Multivariable HRs of incident CVD per additional live birth are shown in Table 2 for both sexes. When live birth was adjusted with age in model 1, each additional live birth was associated with HRs of 1.07 [CI: 1.03–1.12] for women and 1.02 [0.97–1.08] for men, which were significant among women. However, after further adjustment in model 2, the HRs for each additional live births reached 1.04 [0.99–1.09] among women and 1.01 [0.95–1.07] among men.

**Table 2:** Multivariable hazard ratios (HR) and 95% confidence intervals (CI) of incident CVD per additional live birth among women and men: Tehran Lipid and Glucose Study, Iran, 1999–2016

	Women				Men			
	Model 1		Model 2		Model 1		Model 2	
	HR (95% CI)	***p***-value	HR (95% CI)	***p***-value	HR (95% CI)	***p***-value	HR (95% CI)	***p***-value
**Live birth (per each additional)**	1.07 (1.03–1.12)	0.002	1.04 (0.99–1.09)	0.094	1.02 (0.97–1.08)	0.474	1.01 (0.95–1.07)	0.714
**Age (year)**	1.07 (1.06–1.08)	< 0.001	1.05 (1.03–1.06)	< 0.001	1.06 (1.05–1.07)	< 0.001	1.06 (1.05–1.07)	< 0.001
**BMI (kg/m**^**2**^**)**			1.01 (0.99–1.03)	0.404			1.01 (0.99–1.04)	0.219
**T2DM, yes**			2.37 (1.92–2.91)	< 0.001			2.41 (1.92–3.01)	< 0.001
**Hypertension, yes**			1.84 (1.49–2.26)	< 0.001			1.55 (1.28–1.88)	< 0.001
**Hypercholesterolemia, yes**			1.82 (1.38–2.41)	< 0.001			1.49 (1.24–1.79)	< 0.001
**Low physical activity, yes**			1.12 (0.91–1.39)	0.282			1.03 (0.85–1.26)	0.735
**Educational levels**								
Illiterate/primary school			Reference				Reference	
Below diploma/diploma			0.78 (0.59–1.03)	0.084			1.14 (0.92–1.41)	0.229
Above diploma			0.67 (0.34–1.32)	0.248			0.96 (0.71–1.29)	0.772
**Smoking status**								
Never			Reference				Reference	
Former			1.09 (0.71–1.69)	0.693			1.27 (1.01–1.59)	0.044
Current			1.65 (1.09–2.50)	0.018			1.60 (1.30–1.96)	< 0.001
**FH of premature CVD, yes**			1.61 (1.31–1.99)	< 0.001			1.32 (1.04–1.68)	0.023
**History of miscarriage, yes**			1.18 (0.98–1.42)	0.088				
**OCP use, yes**			0.48 (0.20–1.18)	0.109				

*BMI* Body mass index, *T2DM* Type 2 diabetes mellitus, *OCP* Oral contraceptive pill, *CVD* Cardiovascular disease, *FH* Family history

Model 1: adjusted for age

Model 2: for women: adjusted for age, BMI, T2DM, hypertension, hypercholesterolemia, educational levels, smoking status, family history of premature CVD, history of miscarriage, and OCP use; for men: adjusted for age, BMI, T2DM, hypertension, hypercholesterolemia, educational levels, smoking status, and family history of premature CVD

Compared to women with 1 live birth in model 1, women with ≥4 live births had an age-adjusted HR of 2.17 [1.18–4.00]; after more adjustment in model 2, the corresponding HR reached 1.72 [0.92–3.21, *P*-value = 0.087]. Among men, compared to those with 1 live birth, men with 2, 3, and ≥ 4 live births had a higher risk of incident CVD; the risks were significant in the full-adjusted model 2 by HRs of 1.97 [1.24–3.12], 2.08 [1.31–3.31], 2.08 [1.30–3.34], respectively. Importantly, beside the number of live births, traditional CVD risk factors including age, T2DM, hypertension, hypercholesterolemia, family history of premature CVD, former smoking (only among men), and current smoking were significantly associated with incident CVD in model 2 (Table 3**)**.

**Table 3:** Multivariable hazard ratios (HR) and 95% confidence intervals (CI) of incident CVD according to the number of live births among women and men: Tehran Lipid and Glucose Study, Iran, 1999–2016

	Women				Men			
	Model 1		Model 2		Model 1		Model 2	
	HR (95% CI)	***p***-value	HR (95% CI)	***p***-value	HR (95% CI)	***p***-value	HR (95% CI)	***p***-value
**Number of Live births**								
1	Reference		Reference		Reference		Reference	
2	0.89 (0.44–1.80)	0.752	0.86 (0.43–1.73)	0.676	2.00 (1.26–3.16)	0.003	1.97 (1.24–3.12)	0.004
3	1.57 (0.83–2.97)	0.162	1.42 (0.75–2.70)	0.279	2.25 (1.42–3.58)	0.001	2.08 (1.31–3.31)	0.002
≥ 4	2.17 (1.18–4.00)	0.012	1.72 (0.92–3.21)	0.087	2.20 (1.37–3.52)	0.001	2.08 (1.30–3.34)	0.002
**Age (year)**	1.07 (1.06–1.08)	< 0.001	1.04 (1.03–1.06)	< 0.001	1.05 (1.04–1.06)	< 0.001	1.05 (1.04–1.06)	< 0.001
**BMI (kg/m**^**2**^**)**			1.01 (0.98–1.03)	0.613			1.01 (0.99–1.04)	0.270
**T2DM, yes**			2.37 (1.93–2.91)	< 0.001			2.37 (1.89–2.96)	< 0.001
**Hypertension, yes**			1.83 (1.49–2.25)	< 0.001			1.56 (1.29–1.90)	< 0.001
**Hypercholesterolemia, yes**			1.80 (1.37–2.38)	< 0.001			1.47 (1.22–1.77)	< 0.001
**Low physical activity, yes**			1.13 (0.91–1.40)	0.266			1.04 (0.85–1.26)	0.728
**Educational levels**								
Illiterate/primary school			Reference				Reference	
Below diploma/diploma			0.86 (0.64–1.14)	0.284			1.14 (0.93–1.41)	0.211
Above diploma			0.79 (0.40–1.57)	0.503			0.96 (0.71–1.29)	0.771
**Smoking status**								
Never			Reference				Reference	
Former			1.11 (0.71–1.71)	0.653			1.27 (1.01–1.60)	0.039
Current			1.68 (1.11–2.55)	0.014			1.60 (1.30–1.97)	< 0.001
**FH of premature CVD, yes**			1.62 (1.32–2.00)	< 0.001			1.33 (1.05–1.69)	0.020
**History of miscarriage, yes**			1.17 (0.98–1.41)	0.090				
**OCP use, yes**			0.50 (0.20–1.22)	0.126				

*BMI* Body mass index, *T2DM* Type 2 diabetes mellitus, *OCP* Oral contraceptive pill, *CVD* Cardiovascular disease, *FH* Family history

Model 1: adjusted for age

Model 2: for women: adjusted for age, BMI, T2DM, hypertension, hypercholesterolemia, educational levels, smoking status, family history of premature CVD, history of miscarriage, and OCP use; for men: adjusted for age, BMI, T2DM, hypertension, hypercholesterolemia, educational levels, smoking status, and family history of premature CVD

Moreover, we conducted a similar analysis among men aged ≥45 years, who had a high probability of having completed family size (the average number of live births was 3.11 in men aged ≥30 years vs. 4.07 for men aged ≥45 years). Similar to the men aged ≥30 years, among men older than 45 years, having ≥2 live births was associated with an increased CVD risk, with similar effect sizes, although the results did not reach a significant level (Supplementary Table 1). On the other hand, there was no difference between the average number of live birth in women aged ≥30 years (2.46) vs. aged ≥45 years (2.43).

Each unit increase in parity had an age-adjusted HR of 1.08 [1.04–1.13] among women in model 1; the HR remained at significant levels (1.05 [1.01–1.10]), even after further adjustment in model 2 (Table 4**)**. Compared to women with 1 parity, the age-adjusted HRs were 0.97 [0.47–2.00] and 1.41 [0.72–2.76] among those with 2 and 3 parity, respectively. Moreover, having ≥4 parity had an age-adjusted HR of 2.34 [1.24–4.43], and after further adjustment in model 2, it also remained marginally significant (1.86 [0.97–3.56], *P*-value: 0.061). Importantly, beside the number of parity, traditional CVD risk factors including age, T2DM, hypertension, hypercholesterolemia, family history of premature CVD, and current smoking were significantly associated with incident CVD in model 2 (Table 5**)**.

**Table 4:** Multivariable hazard ratios (HR) and 95% confidence intervals (CI) of incident CVD per additional parity among women: Tehran Lipid and Glucose Study, Iran, 1999–2016

	Model 1		Model 2	
	HR (95% CI)	***p***-value	HR (95% CI)	***p***-value
**Parity (per each additional)**	1.08 (1.04–1.13)	< 0.001	1.05 (1.01–1.10)	0.028
**Age (year)**	1.07 (1.06–1.08)	< 0.001	1.05 (1.03–1.06)	< 0.001
**BMI (kg/m**^**2**^**)**			1.01 (0.99–1.03)	0.415
**T2DM, yes**			2.36 (1.92–2.90)	< 0.001
**Hypertension, yes**			1.84 (1.49–2.26)	< 0.001
**Hypercholesterolemia, yes**			1.82 (1.38–2.41)	< 0.001
**Low physical activity, yes**			1.12 (0.91–1.39)	0.285
**Educational levels**				
Illiterate/primary school			Reference	
Below diploma/diploma			0.79 (0.59–1.05)	0.100
Above diploma			0.68 (0.34–1.34)	0.267
**Smoking status**				
Never			Reference	
Former			1.09 (0.71–1.69)	0.686
Current			1.65 (1.09–2.51)	0.017
**FH of premature CVD, yes**			1.62 (1.31–1.99)	< 0.001
**History of miscarriage, yes**			1.17 (0.97–1.41)	0.091
**OCP use, yes**			0.48 (0.20–1.18)	0.108

*BMI* Body mass index, *T2DM* Type 2 diabetes mellitus, *OCP* Oral contraceptive pill, *CVD* Cardiovascular disease, *FH* Family history

Model 1: adjusted for age

Model 2: adjusted for age, BMI, T2DM, hypertension, hypercholesterolemia, educational levels, smoking status, family history of premature CVD, history of miscarriage, and OCP use

**Table 5:** Multivariable hazard ratios (HR) and 95% confidence intervals (CI) of incident CVD according to the number of parity among women: Tehran Lipid and Glucose Study, Iran, 1999–2016

	Model 1		Model 2	
	HR (95% CI)	***p***-value	HR (95% CI)	***p***-value
**Number of parity**				
1	Reference		Reference	
2	0.97 (0.47–2.00)	0.931	0.93 (0.45–1.93)	0.855
3	1.41 (0.72–2.76)	0.315	1.32 (0.67–2.60)	0.417
≥ 4	2.34 (1.24–4.43)	0.009	1.86 (0.97–3.56)	0.061
**Age (year)**	1.07 (1.06–1.08)	< 0.001	1.04 (1.03–1.06)	< 0.001
**BMI (kg/m**^**2**^**)**			1.00 (0.98–1.03)	0.647
**T2DM, yes**			2.36 (1.92–2.90)	< 0.001
**Hypertension, yes**			1.82 (1.48–2.24)	< 0.001
**Hypercholesterolemia, yes**			1.80 (1.36–2.38)	< 0.001
**Low physical activity, yes**			1.13 (0.91–1.40)	0.255
**Educational levels**				
Illiterate/primary school			Reference	
Below diploma/diploma			0.87 (0.65–1.15)	0.321
Above diploma			0.79 (0.40–1.58)	0.510
**Smoking status**				
Never			Reference	
Former			1.11 (0.71–1.71)	0.649
Current			1.67 (1.10–2.53)	0.016
**FH of premature CVD, yes**			1.62 (1.32–2.00)	< 0.001
**History of miscarriage, yes**			1.17 (0.97–1.41)	0.092
**OCP use, yes**			0.49 (0.20–1.20)	0.121

*BMI* Body mass index, *T2DM* Type 2 diabetes mellitus, *OCP* Oral contraceptive pill, *CVD* Cardiovascular disease, *FH* Family history

Model 1: adjusted for age

Model 2: adjusted for age, BMI, T2DM, hypertension, hypercholesterolemia, educational levels, smoking status, family history of premature CVD, history of miscarriage, and OCP use

Compared to our main analysis (using follow-up time as the time axis), using age as the time axis yielded slightly lower effect sizes, but the results were overall similar and the 95% CIs overlapped; however, among women, the effect of parity as a continuous variable on incident CVD lost its significance in the sensitive analysis (Supplementary Table 2).

## Discussion

This is the first population-based study conducted in the MENA region which examines the impact of the number of parity/live birth(s) on incident CVD events among both sexes during more than 15 years of follow-up.

Among women, the number of live births has a J-shaped association with incident CVD, with the lowest risk for those with 2 births. After adjustment for a wide series of important risk factors, including age, T2DM, hypertension, hypercholesterolemia, smoking status, and positive history of premature CVD -all of which remained significant risk factors in our analysis - each unit increase in parity was associated with a 5% higher risk of CVD events among women. Moreover, women with ≥4 parity had a more than 80% higher risk for incident CVD (marginally significant). Focusing on men, in comparison with participants who had 1 live birth, those with 2, 3, and ≥ 4 live births had about 100% higher risk for incident CVD in the presence of important traditional risk factors.

The findings of the current study about the association of parity number with incident CVD among women are consistent with a meta-analysis study on this issue by Li et al. [9] They found a non-linear J-shaped dose-response relationship between the number of parity and CVD among women. They also reported that each unit increase in the number of live birth led to a 4% increased risk of incident CVD; however, the authors showed significant high heterogeneity between studies (I^2^ = 89.6%). In another meta-analysis [23], Lv et al. also found a similar J-shaped association between parity number and CVD mortality with the lowest risk for women with 4 live births. They also found that each live birth was associated with a 1% non-significant increased risk for CVD mortality (I^2^ = 86.4%). It should be noted that the significant heterogeneity in these two meta-analyses [9, 23] could be related to different study populations, sample sizes, and other epidemiologic aspects of studies. Marginally significant increased CVD risk for women with ≥4 parity in our results agrees with the findings of other studies [24, 25]. Furthermore, our findings are in line with some previous cohort studies on CVD morbidity and mortality risk assessment, which show a J- or U-shaped association with the lowest risk for women with 2 live births or parity [8, 26]. However, in some others, there is no relation among women across the number of parity/live birth(s) [11, 13, 27], or their significance of association was lost after adjustment for other factors [12, 28, 29].

Although most of the previous studies on the current issue focused only on women, the limited data on men was controversial as well [16]. Similar to the current report, Peters et al. reported that in comparison with Chinese men who had 1 child, men with ≥2 children and men with ≥3 children had a higher risk of incident CHD and stroke, respectively. Among Chinese men, they also showed that each additional child significantly increased the risk of CHD and stroke by 3 and 2%, respectively [17]; however, we did not find a linear relationship between the number of live births and incident CVD among Tehranian men. On the other hand, Eisenberg et al. found a negative association between the number of offspring and CVD mortality. Based on their report, each additional child decreased the risk of CVD mortality by 2% among American men [16]. Furthermore, some previous studies could not detect any significant association between the number of live births and CVD mortality and morbidity among men [30, 31]. However, some others reported J- or U-shaped associations among men, which were similar to their female population study [26, 32].

A possible explanation for the association between parity and incident CVD in women is the biologic pathway. During pregnancy, some physiologic changes can have adverse effects for incident CVD which remained even after delivery, including weight gain, dyslipidemia, increased plasma glucose and insulin resistance as well as endothelial dysfunction and inflammatory and hemostatic processes [7–9]. In the light of multiple pregnancies, exposure time to these changes increased [9, 33]. This accumulative effect of repeating parity on traditional risk factors might be an important neglected residual confounding factor in the current study and similar researches in this field.

Beside the impact of the biologic pathway, psycho-socio-economic factors were reported to have a potentially important role in the pathway between the number of children and CVD development in both parents [14, 15]. In a previous study on a Swedish population, Barclay et al. compared the effect of the number of live births on CVD mortality between adoptive and biologic parents [14]. They showed that CVD mortality is higher in biological parents than adoptive parents, which means that the biologic pathway had inevitable effects. On the other hand, finding a U-shaped pattern in this study among adoptive male parents suggested that the biological pathway couldn’t be the only explanation for their findings, and the socioeconomic and lifestyle pathway should be considered as another explanation. Moreover, some previous studies [15, 17, 26] have found a similar pattern for men and women in their population study, which confirmed the strong role of socioeconomic and lifestyle pathway. It should be considered that during our study recruitment period, the policy of the Iranian government was based on reducing population growth, so there was minimal economic support for Iranian parents [34]. Furthermore, according to the data of the statistical center of Iran in 2017, household income per capita reduced with increased family size [35]. It means parents with more children might be under financial pressure. These economic problems could have adverse effects on socioeconomic status, leading to CVD development through psychosocial factors (anxiety and depression development), using unhealthier diet, smoking initiation, limiting leisure-time physical activity, poor access to health care, and little knowledge about diseases [36–38]. Since head of the Iranian family is traditionally the father [39], the stronger effect the of number of children among men is expectable through the socioeconomic and life style pathway; the issue needs serious and deep investigations in future studies.

Although we couldn’t find a strong protective role for childbearing in our data set, previous studies suggested that adult children may provide important social and financial support to their parents [14, 40]. These supports might be more important at older ages, where parents often need help for basic daily needs, accessing routine medical care, and prevention of social isolation [40]. Moreover, it was suggested that childbearing could cause developing a healthier lifestyle in parents [14, 40].

In addition to the CVD events, in previous studies, all-cause mortality [40, 41], diabetes [42], different types of cancer [43–45] were also found as medical outcomes influenced by childbearing. Therefore, it is impossible for us to accurately judge the ideal number of children in Tehran society. Moreover, in addition to the physical health of parents and families, other factors such as mental health, socioeconomic status, supports, employment, income, and life satisfaction should also be considered in the determination of the ideal children’s number. Hence, it seems that due to the large differences in the mentioned factors in the community, instead of talking about a fixed value of the ideal number of children for all families, it is better to determine the ideal number of children for each family individually based on a predictive model including all the potential effective factors.

The strengths of the current study consist of addressing the effect of the number of parity/live birth(s) on CVD development among both sexes in the MENA region for the first time, with standardized measurements for assessment of traditional risk factors rather than relying on self-reported data and adjudicated CVD events. The current study was limited in several ways. First, potential risk factors were considered at the time of baseline phases, and possible changes in risk factors as well as the number of parity/live birth(s), were not considered during the follow-up period. Second, the number of participants who hadn’t had children was too low to permit us to compare the impact of nulliparity with ever parity. Third, we did not have access to valid data on participant job status, income, and diet, which can clarify the socioeconomic and lifestyle pathway. Although we considered education status as a socioeconomic determinant, there was no strong relationship between education levels and economic status among the Iranian population [46]. Fourth, some other reproductive factors of female participants such as breastfeeding, age at first and last live birth, age at menarche, and age at natural menopause were found to have roles in CVD development. However, due to lack of data at the baseline recruitment, these reproductive factors were not accessible to us. Finally, our population study included only residents of a metropolitan city and cannot be generalized to rural populations.

## Conclusion

To sum up, a J-shaped association was found between the number of live births and incident CVD among women. Moreover, for CVD events, besides the effects of traditional risk factors, each additional pregnancy increased the risk by 5%. We also found that women with ≥4 parity showed a marginally significant increased risk. Among men, those with only 1 child had the lowest risk of CVD events. It can be suggested that the number of parity/live birth(s) is linked to CVD among the Iranian population, with this issue being more prominent among men. Further research is needed to support our results and clarify the pathways between the number of parity/live birth(s) and CVD development among the Iranian population by considering potential factors, particularly psycho-socio-economic factors.

## Supplementary Information

**Additional file 1: Table S1.** Multivariable hazard ratios (HR) and 95% confidence intervals (CI) of incident CVD according to the number of live births among men aged ≥45 years: Tehran Lipid and Glucose Study, Iran, 1999–2016.

**Additional file 2: Table S2.** Multivariable hazard ratios (HR) and 95% confidence intervals (CI) of incident CVD, comparing the use of follow-up time and age as axis for the Cox model: Tehran Lipid and Glucose Study, Iran, 1999–2016.

## Data Availability

The datasets used and/or analysed during the current study are available from the corresponding author on reasonable request.

## Abbreviations

GBD: Global Burden of Disease
CVD: Cardiovascular Disease
CHD: Coronary Heart Disease
MENA: Middle East and North Africa
TLGS: Tehran Lipid and Glucose Study
BMI: Body Mass Index
FPG: Fasting Plasma Glucose
TC: Total Cholesterol
SBP: Systolic Blood Pressure
DBP: Diastolic Blood Pressure
OCP: Oral Contraceptive Pill
BP: Blood Pressure
HDL-C: High-density Lipoprotein Cholesterol
TG: Triglycerides
MI: Myocardial Infarction
ECG: Electrocardiogram
T2DM: Type 2 Diabetes Mellitus
LRC: Lipid Research Clinic
MAQ: Modifiable Activity Questionnaire
MET: Metabolic Equivalent Task-minutes per Week
HR: Hazard ratio
95% CI: 95% Confidence Interval
IQR: Interquartile Range
