# Supplementary material for: Number of parity/live birth(s) and cardiovascular disease among Iranian women and men: results of over 15 years of follow-up
Source: BMC Pregnancy Childbirth. 2021 Jan 7;21:28. doi: 10.1186/s12884-020-03499-2 (PMC7792076; doi:10.1186/s12884-020-03499-2)
Supplement: Supplementary file 1 — Additional file 1: Table S1. Multivariable hazard ratios (HR) and 95% confidence intervals (CI) of incident CVD according to the number of live births among men aged ≥45 years: Tehran Lipid and Glucose Study, Iran, 1999–2016. [file 12884_2020_3499_MOESM1_ESM.docx]

| **Supplementary Table 1: Multivariable hazard ratios (HR) and 95% confidence intervals (CI) of incident CVD according to the number of live births among men aged ≥45 years: Tehran Lipid and Glucose Study, Iran, 1999-2016.** | | | | |
| --- | --- | --- | --- | --- |
|  | **Model 1** | | **Model 2** | |
|  | **HR (95% CI)** | **p-value** | **HR (95% CI)** | **p-value** |
| **Live birth (per each additional)** | 1.01 (0.96-1.07) | 0.616 | 1.01 (0.95-1.07) | 0.807 |
| **Number of Live births** |  |  |  |  |
| - **1** | Reference |  | Reference |  |
| - **2** | 1.89 (0.81-4.44) | 0.143 | 1.80 (0.77-4.24) | 0.176 |
| - **3** | 2.10 (0.92-4.80) | 0.077 | 1.97 (0.86-4.50) | 0.108 |
| - **≥ 4** | 2.10 (0.94-4.72) | 0.072 | 2.03 (0.90-4.58) | 0.087 |
|  |  | |  | |
| CVD: cardiovascular disease.  Model 1: adjusted for age.  Model 2: adjusted for age, body mass index, type 2 diabetes mellitus, hypertension, hypercholesterolemia, educational level, smoking status, and family history of premature CVD. | | | | |
