# Supplementary material for: Number of parity/live birth(s) and cardiovascular disease among Iranian women and men: results of over 15 years of follow-up
Source: BMC Pregnancy Childbirth. 2021 Jan 7;21:28. doi: 10.1186/s12884-020-03499-2 (PMC7792076; doi:10.1186/s12884-020-03499-2)
Supplement: Supplementary file 2 — Additional file 2: Table S2. Multivariable hazard ratios (HR) and 95% confidence intervals (CI) of incident CVD, comparing the use of follow-up time and age as axis for the Cox model: Tehran Lipid and Glucose Study, Iran, 1999–2016. [file 12884_2020_3499_MOESM2_ESM.docx]

| **Supplementary Table 2. Multivariable hazard ratios (HR) and 95% confidence intervals (CI) of incident CVD, comparing the use of follow-up time and age as axis for the Cox model: Tehran Lipid and Glucose Study, Iran, 1999-2016.** | | | | |
| --- | --- | --- | --- | --- |
|  | **Women** | | **Men** | |
|  | **Follow-up time as time axis**  **Adjusted HR (95% CI)^⁎^** | **Age as time axis**  **Adjusted HR (95% CI)^⁑^** | **Follow-up time as time axis**  **Adjusted HR (95% CI)^⁎^** | **Age as time axis**  **Adjusted HR (95% CI)^⁑^** |
| **Live birth (per each additional)** | 1.04 (0.99-1.09) | 1.03 (0.98-1.08) | 1.01 (0.95-1.07) | 1.00 (0.94-1.06) |
| **Number of Live births** |  |  |  |  |
| - 1 | Reference | Reference | Reference | Reference |
| - 2 | 0.86 (0.43-1.73) | 0.87 (0.43-1.75) | 1.97 (1.24-3.12) | 1.67 (1.05-2.67) |
| - 3 | 1.42 (0.75-2.70) | 1.20 (0.63-2.26) | 2.08 (1.31-3.31) | 1.63 (1.01-2.62) |
| - ≥ 4 | 1.72 (0.92-3.21) | 1.35 (0.73-2.49) | 2.08 (1.30-3.34) | 1.62 (1.01-2.60) |
|  |  |  |  |  |
| **Parity (per each additional)** | 1.05 (1.01-1.10) | 1.04 (0.99-1.08) |  |  |
| **Number of parity** |  |  |  |  |
| - 1 | Reference | Reference |  |  |
| - 2 | 0.93 (0.45-1.93) | 0.93 (0.45-1.92) |  |  |
| - 3 | 1.32 (0.67-2.60) | 1.11 (0.57-2.19) |  |  |
| - ≥ 4 | 1.86 (0.97-3.56) | 1.44 (0.76-2.73) |  |  |
| ⁎ For women: the model adjusted for age, BMI, T2DM, hypertension, hypercholesterolemia, educational status, smoking status, family history of premature CVD, history of miscarriage, and OCP use; for men: the model adjusted for age, BMI, T2DM, hypertension, hypercholesterolemia, educational status, smoking status, and family history of premature CVD.  ⁑ for women: The model adjusted for BMI, T2DM, hypertension, hypercholesterolemia, educational status, smoking status, family history of premature CVD, history of miscarriage, and OCP use; for men: the model adjusted for BMI, T2DM, hypertension, hypercholesterolemia, educational status, smoking status, and family history of CVD.  CVD: cardiovascular disease; BMI: body mass index; T2DM: type 2 diabetes mellitus; OCP: oral contraceptive pill. | | | | |
